# Coffee consumption is not associated with ovarian cancer risk: a dose-response meta-analysis of prospective cohort studies

**DOI:** 10.18632/oncotarget.24829

**Published:** 2018-04-17

**Authors:** Massimiliano Berretta, Agnieszka Micek, Alessandra Lafranconi, Sabrina Rossetti, Raffaele Di Francia, Paolo De Paoli, Paola Rossi, Gaetano Facchini

**Affiliations:** ^1^ Department of Medical Oncology, National Cancer Institute-IRCCS, Aviano, Italy; ^2^ Department of Epidemiology and Population Studies, Jagiellonian University Medical College, Krakow, Poland; ^3^ The Research Centre on Public Health, University Milano-Bicocca, Milan, Italy; ^4^ Departmental Unit of Experimental Uro-Andrological Clinical Oncology, Department of Uro-Gynecological Oncology, Istituto Nazionale Tumori Fondazione G. Pascale-IRCCS, Naples, Italy; ^5^ Hematology-Oncology and Stem Cell Transplantation Unit, National Cancer Institute, Fondazione G. Pascale IRCCS, Naples, Italy; ^6^ Scientific Directorate, National Cancer Institute, Aviano, Italy; ^7^ Department of Biology and Biotechnology, L. Spallanzani University of Pavia, Pavia, Italy

**Keywords:** coffee, ovarian cancer, postmenopausal, meta-analysis, cohort studies

## Abstract

**Background:**

Coffee consumption has been associated with numerous cancers, but evidence on ovarian cancer risk is controversial. Therefore, we performed a meta-analysis on prospective cohort studies in order to review the evidence on coffee consumption and risk of ovarian cancer.

**Methods:**

Studies were identified through searching the PubMed and MEDLINE databases up to March 2017. Risk estimates were retrieved from the studies, and dose-response analysis was modelled by using restricted cubic splines. Additionally, a stratified analysis by menopausal status was performed.

**Results:**

A total of 8 studies were eligible for the dose-response meta-analysis. Studies included in the analysis comprised 787,076 participants and 3,541 ovarian cancer cases. The results showed that coffee intake was not associated with ovarian cancer risk (RR = 1.06, 95% CI: 0.89, 1.26). Stratified and subgroup analysis showed consisted results.

**Conclusions:**

This comprehensive meta-analysis did not find evidence of an association between the consumption of coffee and risk of ovarian cancer.

## INTRODUCTION

Coffee is among the most consumed beverages worldwide: in Europe, the geographic area showing the highest coffee consumer in the world, people are reported to drink 725 million cups of coffee every day [1]. In light of its wide consumption, it is an important public health goal to assess whether coffee has protective or detrimental effects against cancer risk.

Current evidence from epidemiological and experimental studies suggests that coffee consumption may exert beneficial effects towards non-communicable diseases [2]. In particular, coffee consumption has been associated with decreased risk of cardio-metabolic conditions, including metabolic syndrome [3], diabetes [4] and cardiovascular disease [5]. Research on cancer risk showed more controversial findings: a summary of evidence reported that coffee could have a protective role against the development of various cancers, including liver, colorectal, endometrial, oral, melanoma, and prostate cancer among others [6–12]. However, other revision of existing literature reported contrasting results [13, 14]: this conclusion was based on a dose-response meta-analysis of 10 cohort studies, and the relative risk (RR) for an increment of 1 cup/day appeared to be not significantly associated with risk of overall cancers [15]. Numerous confounders and effect modifiers can have a role in assessing the relationship between coffee consumption and health outcomes: for instance, recent meta-analyses on coffee consumption and various health outcomes pointed out that the ambiguous results on cancer mortality and hypertension were due to the modifying effect of smoking [16, 17].

Ovarian cancer has a major epidemiological and social burden for women worldwide: in 2012, women with a diagnosis of ovarian cancer occurred in the last 5 years were over 587,000 and 157,000 worldwide and in Europe, respectively [18, 19]. In 2013, deaths accounted for ovarian cancer were about 158,000 worldwide, together with over 4 million disability adjusted life years (DALYs); such figure significantly increased from 1990 (2.7 million DALYs) and poses ovarian cancer as the worldwide 12th contributor of death and disability among cancers [20].

The evidence on the association between coffee drinking and the risk of ovarian cancer is quite limited and inconsistent. Results of a meta-analysis of prospective cohort studies revealed a non-significant positive association between coffee drinking and risk of ovarian cancer [21]. However, more recent data has been published and comprehensive summary of evidence is lacking. Thus, the aim of this study is to update current evidence on the association between dietary coffee consumption and risk of ovarian cancer testing also the dose-response relation.

## METHODS

We performed a meta-analysis of prospective cohort studies following Meta-Analysis of Observational Studies in Epidemiology (MOOSE) protocols throughout design, execution, analysis and reporting stages (Supplementary Table 1).

### Search strategy

To collect and critically review the evidence, we performed a comprehensive literature search in PubMed (http://www.ncbi.nlm.nih.gov/pubmed/) and EMBASE (http://www.embase.com/) databases, from the earliest available online indexing year to March 2017. The search was limited to studies published in English. Search terms included the following: (coffee OR caffeine OR beverages) AND (ovarian) AND (cancer OR carcinoma OR neoplasm) (Supplementary Table 2). Titles and abstracts of all identified studies were screened by two members of the team. Studies were eligible for inclusion in the meta-analysis if they met the following inclusion criteria: 1) the study had a prospective design; 2) the exposure of interest was coffee consumption; 3) the outcome was ovarian cancer incidence; 4) the investigators reported relative risks with 95% confidence intervals for 3 or more quantitative categories of coffee consumption. When discrepancies over inclusion arose, inclusion criteria were assessed to reach a consensus. Reference lists of included manuscripts were also screened for any additional study not previously identified. When duplicate reports on the same cohort were identified, we included the one that provided the largest number of cases (or entire cohort) or with the longest follow-up for each endpoint of interest.

### Data extraction

Data were abstracted from all identified studies using a standardized extraction form. Information was extracted from each study and consisted of: 1) first author name; 2) year of publication; 3) study cohort name; 4) country; 5) number of participants; 6) gender of participants; 7) age range of the study population at baseline; 8) categories of consumption; 9) coffee type; 10) follow-up period; 11) endpoints and cases; 12) distributions of cases and person-years, HRs, and 95% CIs for all categories of exposure; 13) covariates used in adjustments. Extraction of data was performed independently by two authors. Discrepancies were discussed and resolved by consensus. The quality of included studies was assessed according to the Newcastle-Ottawa Quality Assessment Scale [22].

### Statistical analysis

Highest *versus* lowest and dose-response meta-analyses were performed to determine the relationship between coffee intake and ovarian cancer risk. The most fully adjusted RRs/HRs with 95% CI for all categories of exposure were extracted. Random-effects models were used to calculate pooled effects, wherein HRs were deemed as equivalent to relative risks (RRs) [23]. Heterogeneity was assessed through the *I*^*2*^ statistic, which estimates the fraction of variance that is due to heterogeneity and by *Q* test. The level of significance for the *Q* test was defined as *P* < 0.10. The values of *I*^*2*^ statistic ≤25%, 25–50%, 50–75%, and >75% indicated no, small, moderate, and significant heterogeneity, respectively. The stability of the results and potential sources of heterogeneity were examined in a sensitivity analysis excluding one study at a time and in subgroup analysis according to menopausal status and geographical area. Included studies did not provide datasets of stratified analysis to test for potential confounders/effect modifiers, such as smoking or BMI. Therefore, subgroup analyses were only performed according to adjustment for smoking status, BMI, education level and alcohol consumption. Publication bias was evaluated by a visual investigation of funnel plots for potential asymmetry.

To examine linear and non-linear relationship between coffee intake and risk of ovarian cancer, random-effects dose-response meta-analysis was used. We extracted data on the amount of coffee intake, distributions of cases and person-years (when available), and RRs/HRs with 95% CIs for ≥3 exposure categories. In each study across categories of consumption, the mean or median intake of coffee was assigned to the corresponding RR/HR with the 95% CI. When originally in article the range of coffee intake was reported , the midpoint of the range was selected . When the highest category was open ended, we assumed the width of the category to be the same as the adjacent category. When the lowest category was open ended, we set the lower boundary to zero. Random-effects dose-response meta-analysis was performed in two-stages. In the first stage the method reported by Greenland and Orsini (that is, GLS) was used to calculate study-specific slopes (generalized least-squares, GLS) on the basis of results across categories of coffee intake taking into account the correlation within each set of retrieved HRs [24, 25]. Model of restricted cubic splines with 3 knots at fixed percentiles (25%, 50%, and 75%) of the distribution was applied in non-linear dose-response. [26]. We combined the coefficients that had been calculated within each study by performing random-effects meta-analysis with DerSimonian and Laird estimator of between study variance (in linear dose-response meta-analysis) or estimator received by using the method of moments (in non-linear dose-response meta-analysis). We calculated an overall *P*-value by testing that the 2 regression coefficients were simultaneously equal to zero. We then calculated a *P*-value for non-linearity by testing that the coefficient of the second spline was equal to zero. All analyses were performed with R software version 3.0.3 and we mainly used dosresmeta package (Development Core Team, Vienna, Austria).

## RESULTS

### Study characteristics

**Figure 1:**
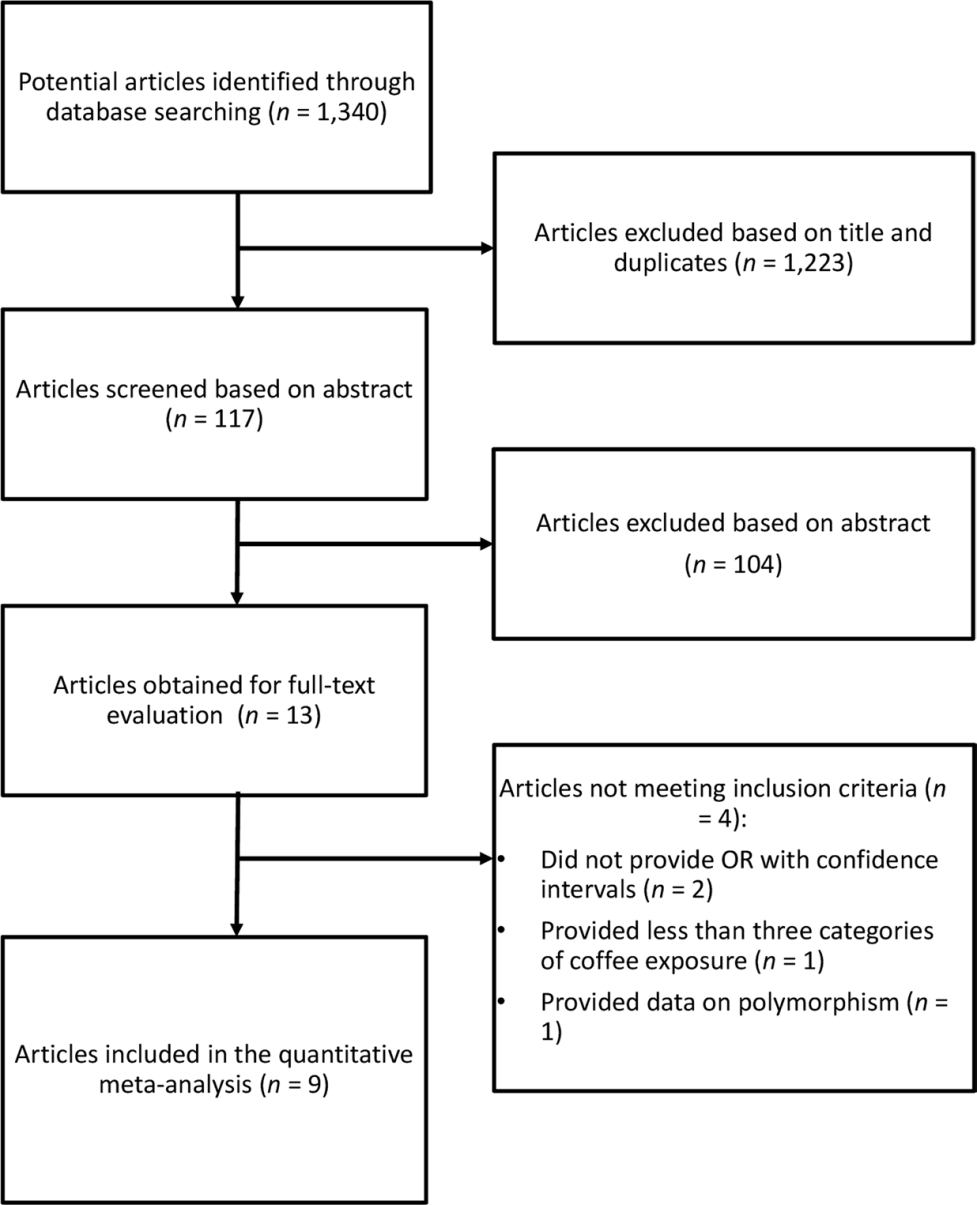
Flow chart and process selection of relevant studies exploring the association between coffee consumption and ovarian cancer risk

We identified a total of 1,340 studies, of which 1,223 were excluded after review of title, and 104 on the basis of abstract (Figure 1). Of the remained thirteen publications, four were not included for the following reasons: 1) the article did not provide relative risks (or similar risk measures) and corresponding confidence intervals; 2) the study provided less than three exposures of coffee consumption; 3) the article reported on gene polymorphism. For the analysis on the association between coffee consumption and ovarian cancer risk, nine studies were eligible [13, 27–34], of which one was a multi-center study [27]. Studies included in the analysis comprised 787,076 participants and 3,541 ovarian cancer cases. Five studies were conducted in Europe [27, 28, 30, 31, 33] and four in USA [13, 29, 32, 34]. Three studies provided risk estimates for postmenopausal status [13, 29, 33], and three for caffeinated and decaffeinated coffee consumption (27, 29, 34). The follow-up in prospective studies ranged from about 11 to 24 years, and the age range at study baseline was 25-76 years. Characteristics of the studies included in the meta-analysis are provided in Table 1.

**Table 1:** Characteristics of the cohort studies included in the meta-analysis

Author, year	Cohort name, country	Years of study, follow-up	Cases; total population	Age range	Adjustments
Larsson 2005	SMC, Sweden	1987–2004, 15.1y (mean)	301; 61,057	40–76y	Age in months, BMI, education, parity, oral contraceptive use, and intakes of total energy, fruit, vegetables, milk, and tea.
Silvera 2007	NBSS, Canada	1980–2000, 16.4y (mean)	264; 48,776	40–59y	Age, smoking history, pack-years of smoking, alcohol intake, education, BMI, parity, participation in vigorous physical activity, menopausal status, oral contraceptive use, energy intake, lactose intake, study center, and randomization group.
Steevens 2007	NLCS, Netherlands	1986–1999, 13.3y	280; 62,573	55–69y	Age, use of oral contraceptives, parity, cigarette smoking, tea.
Lueth 2008	IWHS, USA	1986–2004, 18y (maximum)	266; 29,060	55–69y	Age, smoking, BMI, age at menopause, parity, oral contraceptive use, education level, physical activity, and total energy intake.
Tworoger 2008	NHS, USA	1980–2004, 24y (maximum)	507; 80,253	30–55y	Age, parity, oral contraceptive use, postmenopausal hormone use, tubal ligation, smoking status, and BMI.
Nilsson 2010	VIP, Sweden	1985–2007, 15y (maximum)	71; 32,178	30–60y	Age, sex, BMI, smoking, education, recreational physical activity.
Braem 2012	EPIC, Europe	1992–NA; 11.7y (median)	1,244; 330,849	25–70y	Center, age, parity, oral contraceptive use, BMI, smoking status, alcohol consumption, total energy intake, duration of breastfeeding, menopausal status, height, educational level.
Hashibe 2015	PLCO, USA	1992–2011; 13y (maximum)	162; 50,563	55–74y	Age, sex, race, education, smoking status, smoking frequency, smoking duration, time since stopping smoking for past smokers, and drinking frequency.
Lukic 2016	NOWAC, Norway	1991–2013; 13.1y (average)	446; 91,767	30–70y	Menopausal status at baseline, smoking status, age at smoking initiation, number of pack-years smoked, duration of education, BMI, physical activity level, use of oral contraceptives, alcohol consumption, number of children, use of hormone replacement therapy, and maternal history of breast cancer.

Summary relative risk for highest versus lowest category of coffee consumption

**Figure 2:**
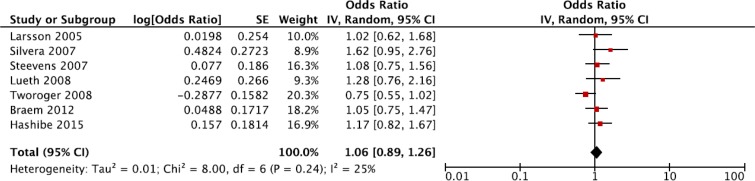
Forest plot of summary relative risks (RRs) of ovarian cancer for the highest versus lowest (reference) category of coffee consumption

The summary risk of ovarian cancer for highest *versus* lowest category of coffee consumption was RR = 1.06, 95% CI: 0.89, 1.26, with no evidence of heterogeneity *I*^*2*^ = 25%, *P* = 0.24 (Figure 2). No publication bias was found after visual inspection of funnel plot (Supplementary Figure 1) Two cohorts, NOWAC and VIP were excluded from the main analysis, as part of their cases are included in the multicentre study EPIC. However, an alternative analysis was performed by including this cohorts and excluding EPIC study; the relative risk was RR = 1.05, 95% CI: 0.88, 1.26; *I*^*2*^ = 20%, *P* = 0.27. Similarly, when taking into account menopausal status, no association between coffee consumption and ovarian cancer risk was found, and RR = 1.15, 95% CI: 0.92, 1.45; *I*^*2*^ = 0%, *P* = 0.87 (Table 2). Finally, no significant differences were found for coffee type or adjustment for potential confounders (Table 2).

**Table 2:** Subgroup analyses of studies reporting risk of ovarian cancer for the highest *versus* lowest (reference) category coffee consumption

Subgroup	No. of datasets	RR (95% CI)	*I*^2^	*P*_heterogeneity_
Total	7	1.06 (0.89, 1.26)	25%	0.24
Geographical area				
North America	4	1.11 (0.79, 1.56)	62%	0.05
Europe	3	1.05 (0.85, 1.32)	0%	0.98
Menopausal status				
Postmenopausal	3	1.15 (0.92, 1.45)	0%	0.87
Premenopausal	0	NA	NA	NA
Coffee type				
Caffeinated	3	1.09 (0.70, 1.68)	78%	0.01
Decaffeinated	3	0.89 (0.66, 1.20)	0%	0.99
Adjusted for smoking				
No	1	1.02 (0.62, 1.69)	NA	NA
Yes	6	1.07 (0.88, 1.31)	37%	0.16
Adjusted for BMI				
No	2	1.13 (0.87, 1.45)	0%	0.76
Yes	5	1.05 (0.81, 1.36)	46%	0.12
Adjusted for education				
No	2	0.89 (0.62, 1.27)	55%	0.14
Yes	5	1.17 (0.97, 1.41)	0%	0.69
Adjusted for alcohol intake				
No	4	0.96 (0.76, 1.22)	26%	0.26
Yes	3	1.18 (0.95, 1.47)	0%	0.40

### Dose-response meta-analysis

Eight studies [13, 28–34] were included in dose-response meta-analysis of prospective studies on coffee consumption and ovarian cancer risk, three of which provided risk estimates for postmenopausal individuals [13, 29, 33].

**Figure 3:**
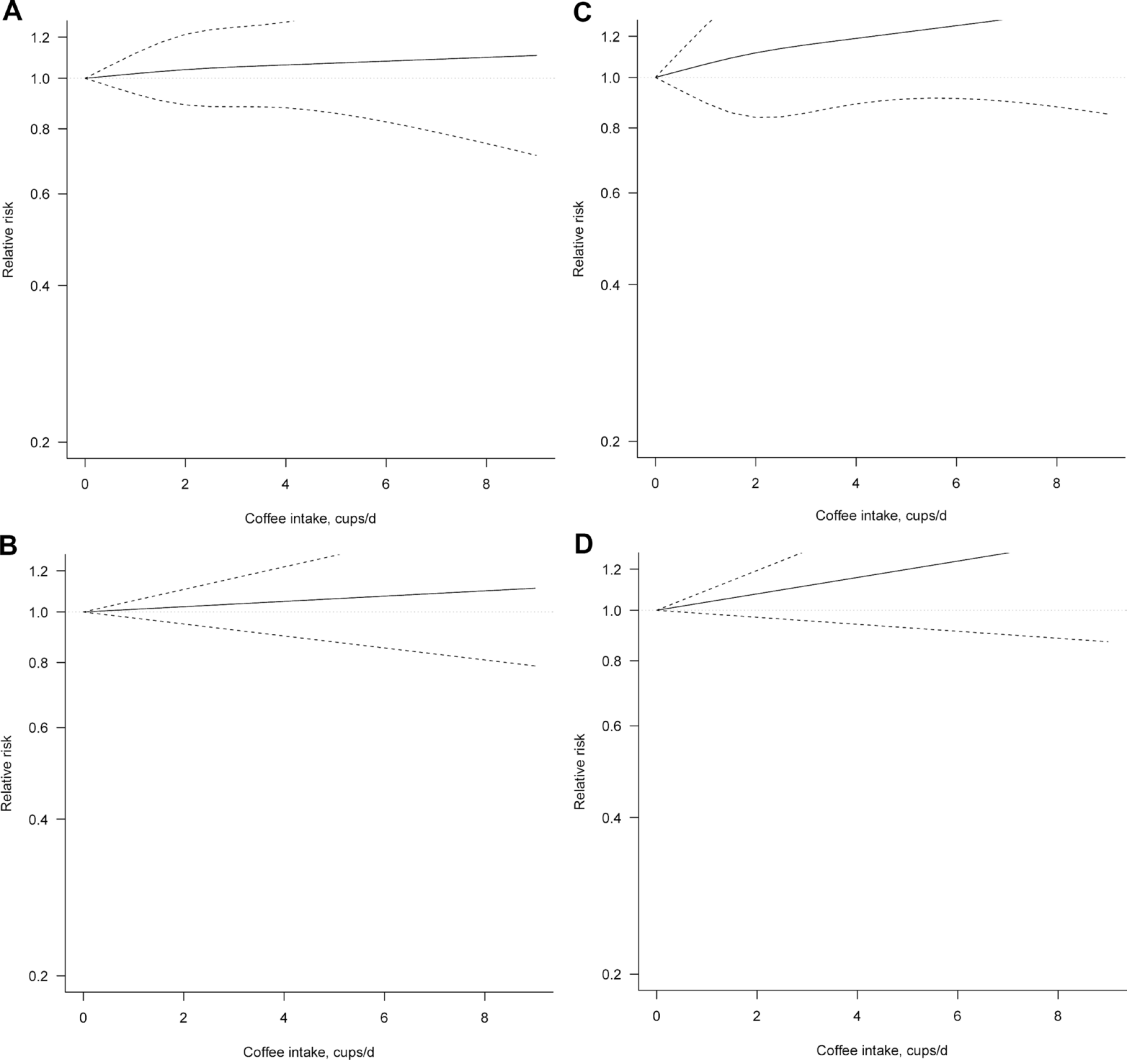
Dose-response association between coffee consumption and ovarian cancer risk (**A**) non-linear, total analysis; (**B**) linear, total analysis (**C**) non-linear, postmenopausal; (**D**) linear, postmenopausal.

We found no evidence of association between coffee consumption and ovarian cancer risk in both analysis on total group of women (Figure 3, Table 3) and when considering only postmenopausal individuals.

**Table 3:** Dose-response meta-analysis of prospective cohort studies on coffee consumption and ovarian cancer risk

	No. of datasets (no. of studies)	Coffee intake (cups/day)								*I*^2^ (%)	*P*_heterogeneity_	*P*_non-linearity_
		0	1	2	3	4	5	6	7			
Total analysis												
Non-linear	8 (8)	Ref.	1.02 (0.93, 1.12)	1.04 (0.89, 1.21)	1.05 (0.88, 1.25)	1.06 (0.88, 1.28)	1.07 (0.86, 1.34)	1.08 (0.82, 1.41)	1.09 (0.79, 1.50)	–	0.55	0.85
Linear	8 (8)	Ref.	1.01 (0.97, 1.05)	1.02 (0.95, 1.10)	1.04 (0.92, 1.16)	1.05 (0.90, 1.22)	1.06 (0.88, 1.28)	1.07 (0.85, 1.35)	1.09 (0.83, 1.42)	32.91	0.17	NA
Postmenopausal												
Non-linear	3 (3)	Ref.	1.06 (0.89, 1.26)	1.12 (0.84, 1.48)	1.15 (0.85, 1.56)	1.19 (0.89, 1.59)	1.22 (0.91, 1.64)	1.26 (0.91, 1.73)	1.29 (0.90, 1.86)	–	0.86	0.79
Linear	3 (3)	Ref.	1.04 (0.98, 1.09)	1.08 (0.97, 1.19)	1.12 (0.95, 1.30)	1.16 (0.94, 1.42)	1.20 (0.93, 1.55)	1.24 (0.91, 1.70)	1.29 (0.90, 1.85)	–	0.90	NA

## DISCUSSION

In this large meta-analysis of prospective cohort studies, we did not find any association of consumption of total, caffeinated, and decaffeinated coffee with risk of ovarian cancer. Despite a large number of studies suggested that an inverse relationship of coffee consumption and cancer risks may be mediated by various mechanisms, such as reduction of oxidative stress and DNA damages, detoxification of carcinogens, inhibition of carcinogenesis, and induction of apoptosis [35–38], a clear association with ovarian cancer risk could not be assessed. Coffee contains thousands of bioactive components including polyphenols, caffeine, diterpens and melanoidins, which have been shown to reduce oxidative stress and exert anti-cancerogenic properties [39, 40]. Dietary polyphenol intake has been associated lower risk of certain cancers and mortality in meta-analysis on prospective cohort studies [41, 42]. A protective effect of polyphenols could be exerted through an indirect action and through triggering defence mechanisms, carcinogenic detoxification, and activation or suppression of onco-suppressors and proto-oncogenes, respectively [43–45]. Additionally, it has been demonstrated that coffee intake improves metabolic features, both in women and men, which in turn could affect the association between cardio-metabolic conditions and certain cancers related to impaired metabolism and hormonal homeostasis [3, 46–49]. However, the results of the present meta-analysis showed that it is not likely a direct association between coffee consumption and ovarian cancer. A possible reason related to lack of effects of its polyphenol content may depend on their bioavailability [50]; in fact, it is still questionable which are the polyphenol metabolites reaching the target tissues, what is their amount, and whether they can actually exert any protective effects specifically on the ovarian cells [51].

Another reason for lack of association retrieved is potential confounding effect of other foods. Coffee consumption might be associated with unhealthy habits (i.e., higher alcohol intake. smoking), which in turn could be related to higher risk of cancers due to a synergistic effect of many functional components rather than an individual food or beverage [52]. Moreover, it has been suggested that polyphenols (including those contained in coffee) may be effective against multiple targets in cancer development and progression especially whether in combination with other polyphenols or micronutrients, such as antioxidant vitamins [53, 54]. In turn, the potential beneficial effects of coffee might be enhanced or counteracted by other dietary components, resulting in an overall null association depending on the overall diet rather than coffee alone. However, this hypothesis on the confounding effect of other foods on coffee should result in null results also for the association with other cancers, which in fact is not supported by other meta-analyses showing a decreased risk of certain cancers associated with higher coffee consumption: thus, either the confounding factors are strictly related to ovarian cancer risk or other explanation should be further investigated.

A major strength of our meta-analysis was the inclusion of cohort studies carried out with a prospective design, which implies detailed exposure and covariate assessment before the diagnosis of the outcome of interest (that is, ovarian cancer). Moreover, we performed a dose-response meta-analysis, which scientific value is higher than meta-analyses with exposure classified as high versus low, as it aims to investigate a possible dose-response relationship. However, the results of this study should be considered in light of some limitation. First, no stratified analysis was performed due to lack of available datasets, which prevented us from an in-depth analysis of potential confounders and effect modifiers, such as smoking status or body weight; despite our adjustment for smoking status, BMI index, education level and alcohol exposure, there is still a chance of unmeasured or residual confounding (e.g. menopausal status, that has not been considered in our analysis). Another limitation lies in the exposure assessment phase: the categories of low and high consumption varied across different studies, therefore overall estimates of high consumption might not be perfectly comparable; moreover, we don’t have data on coffee brewing methods, preparation, cup size, and duration of consumption. Such differences, if properly addressed, could yield to significant results in specific exposure subgroups (for instance, when analysing the relationship between tea consumption and ovarian cancer risk, a positive association has been detected for all tea and for black tea, but not for green tea) [55]. Similarly, histological information on cancer-subtype, to differentiate among epithelial cancer histotypes (“high grade serous” versus “endometrioid” versus “clear cell”), included through a stratified analysis, could highlight different pathological pathways and diversified exposure-disease relationships.

In conclusion, the present study provided a robust assessment of the relationship between coffee consumption and ovarian cancer, which, coherently with previously published literature, appears null. Additional prospective cohort studies specifying also subgroup analyses by key variables (i.e., smoking status, type of coffee, etc.) are needed to further improve current knowledge on such topic.

## SUPPLEMENTARY MATERIALS FIGURE AND TABLES





## Abbreviations

EPIC: European Prospective Investigation into Cancer and Nutrition
IWHS: Iowa Women’s Health Study
NBSS: National Breast Screening Study
NHS: The Nurses’ Health Study
NLCS: The Netherlands Cohort Study
NOWAC: The Norwegian Women and Cancer study
PLCO: Prostate, Lung, Colorectal, and Ovarian Cancer Screening Trial
SMC: The Swedish Mammography Cohort
VIP: Västerbotten intervention project cohort
