# Supplementary material for: Coffee consumption is not associated with ovarian cancer risk: a dose-response meta-analysis of prospective cohort studies
Source: Oncotarget. 2018 Apr 17;9(29):20807–15. doi: 10.18632/oncotarget.24829 (PMC5945528; doi:10.18632/oncotarget.24829)
Supplement: Supplementary file 1 [file oncotarget-09-20807-s001.pdf]

Coffee consumption is not associated with ovarian cancer risk: a dose-response meta-analysis of prospective cohort studies

SUPPLEMENTARY MATERIALS

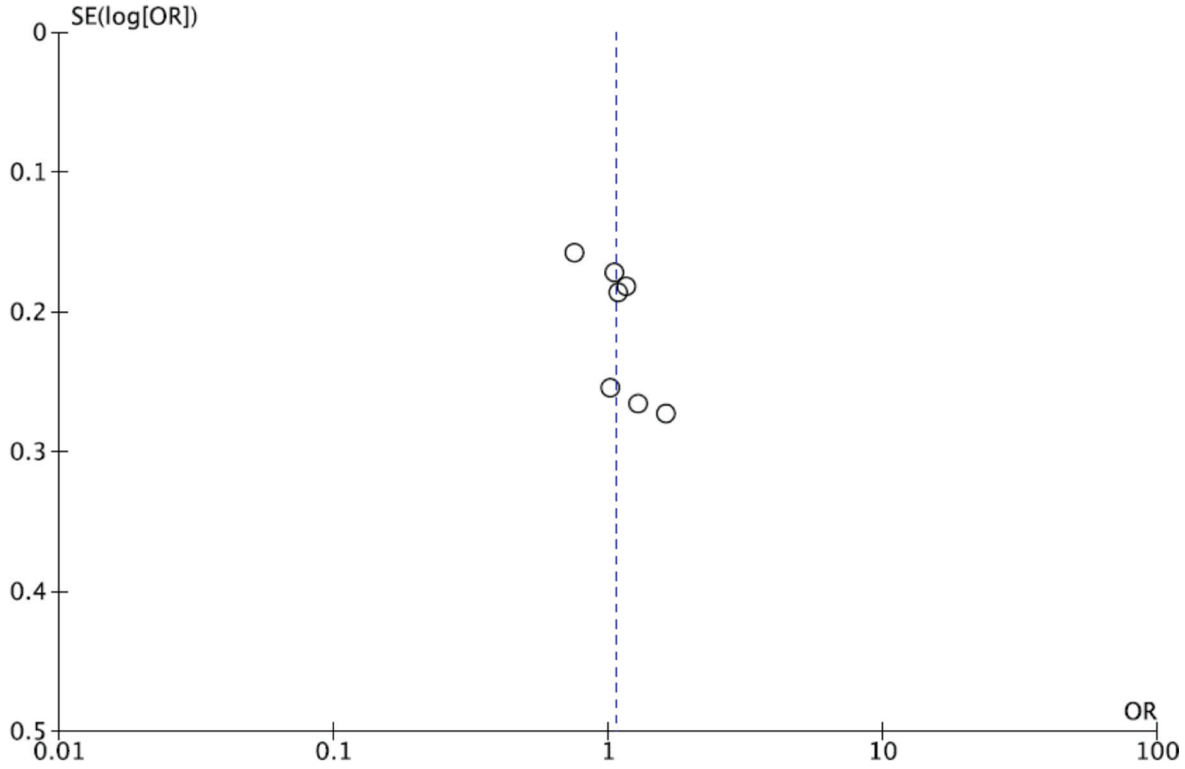

Supplementary Figure 1: Funnel plots for ovarian cancer risk of the highest *versus* lowest (reference) category of coffee consumption.

Supplementary Table 1: Meta-Analysis of observational studies in epidemiology (MOOSE) checklist. See Supplementary_ Table_1

Supplementary Table 2: Search strategy

**Ovarian cancer**	
	(((“coffee”[MeSH Terms] OR “coffee”[All Fields]) OR (“caffeine”[MeSH Terms] OR “caffeine”[All Fields]) OR (“tea”[MeSH Terms] OR “tea”[All Fields]) OR (“beverages”[MeSH Terms] OR “beverages”[All Fields]) OR (“diet”[MeSH Terms] OR “diet”[All Fields]) OR (“diet”[MeSH Terms] OR “diet”[All Fields] OR “dietary”[All Fields])) AND ovarian[All Fields]) AND ((“neoplasms”[MeSH Terms] OR “neoplasms”[All Fields] OR “cancer”[All Fields]) OR (“carcinoma”[MeSH Terms] OR “carcinoma”[All Fields]) OR (“neoplasms”[MeSH Terms] OR “neoplasms”[All Fields] OR “neoplasm”[All Fields]))
